# A Longitudinal Murine Model Reveals Biphasic T Cell Remodeling and Progressive Skeletal Deterioration Under Chronic High-Salt Exposure

**DOI:** 10.3390/cells15090825

**Published:** 2026-05-01

**Authors:** Constanza Quiroga, Santiago Boccardo, Camila M. S. Giménez, Daniela J. Porta, Mercedes Lombarte, Lucas R. Brun, Germán Tirao, Eva V. Acosta Rodríguez, María Angélica Rivoira

**Affiliations:** 1 Instituto de Investigación en Ciencias de la Salud (INICSA), Consejo Nacional de Investigaciones Científicas y Técnicas, Universidad Nacional de Córdoba (CONICET-UNC), Córdoba 5000, Argentina; constanza.quiroga@unc.edu.ar (C.Q.); danielajosefinaporta@gmail.com (D.J.P.); 2 Cátedra de Bioquímica y Biología Molecular, Facultad de Ciencias Médicas, Universidad Nacional de Córdoba, Córdoba 5000, Argentina; 3 Centro de Investigaciones en Bioquímica Clínica e Inmunología (CIBICI), Consejo Nacional de Investigaciones Científicas y Técnicas, Universidad Nacional de Córdoba (CONICET-UNC), Córdoba 5000, Argentina; sboccardo@unc.edu.ar (S.B.); camila_gimenez@unc.edu.ar (C.M.S.G.); eva.acosta@unc.edu.ar (E.V.A.R.); 4 Departamento de Bioquímica Clínica, Facultad de Ciencias Químicas, Universidad Nacional de Córdoba, Córdoba 5000, Argentina; 5 Consejo Nacional de Investigaciones Científicas y Técnicas (CONICET), Ciudad Autónoma de Buenos Aires 1425, Argentina; mercedeslombarte@gmail.com (M.L.); brun@unr.edu.ar (L.R.B.); gtirao@unc.edu.ar (G.T.); 6 Laboratorio de Biología Ósea, Facultad de Ciencias Médicas, Universidad Nacional de Rosario, Rosario 2000, Argentina; 7 Instituto de Física Enrique Gaviola (IFEG), Consejo Nacional de Investigaciones Científicas y Técnicas, Universidad Nacional de Córdoba (CONICET-UNC), Córdoba 5000, Argentina; 8 Facultad de Matemática, Astronomía, Física y Computación (FAMAF), Universidad Nacional de Córdoba (UNC), Córdoba 5000, Argentina

**Keywords:** high-salt diet, bone deterioration, T cell activation, Th17/Treg balance, bone–immune crosstalk, mouse, unilateral nephrectomy

## Abstract

**Highlights:**

**What are the main findings?**

- Chronic high-salt intake induces biphasic T cell remodeling, characterized by early Th17 expansion followed by late suppression of effector activation.
- Sustained sodium overload progressively impairs bone composition, trabecular microarchitecture, and biomechanical strength in both sexes.

**What are the implications of the main findings?**

- A longitudinal murine model integrating immune and skeletal analyses enables time-resolved evaluation of sodium-associated tissue adaptation.

**Abstract:**

Excessive dietary sodium intake has been associated with immune dysregulation, yet its impact on bone health and immune cell dynamics within the bone–immune axis remains poorly understood. We developed a longitudinal murine model to investigate the effects of a high-salt diet (HSD) on bone properties and immunity. Male and female C57BL/6J and Foxp3-GFP mice underwent unilateral nephrectomy and were fed either a normal salt diet (0.2% NaCl) or HSD (4% NaCl) for 20, 60, or 150 days. HSD mice exhibited a transient increase in systolic blood pressure and sustained calciuria without changes in serum calcium or PTH. Progressive impairment of femoral strength and tibial trabecular microarchitecture were observed, along with reduced cortical calcium and phosphorus content. Immune analysis revealed early splenic and bone marrow activation of effector T cells, with increased Th17 and Tc17 populations and a disrupted Th17/Treg balance at 20 days. These changes normalized by 60 days and shifted to suppressed T cell activation at 150 days, suggesting a biphasic immune response. Th17/Treg ratio was associated with bone deterioration. Notably, both sexes showed comparable physiological and immune trends. This integrative model provides a platform to dissect mechanisms linking chronic salt overload, immune dysregulation, and bone fragility.

## 1. Introduction

In recent years, the general population has increased its consumption of ultra-processed foods, rich in sugar and salt, leading to a dietary sodium chloride intake that often exceeds the recommended daily levels [1]. This trend is observed worldwide, regardless of sex, age, ethnicity, or socioeconomic status [2]. Sodium is an essential nutrient when consumed in adequate amounts, playing critical roles in cellular function, osmotic pressure maintenance, acid-base balance, fluid distribution, and key metabolic processes [3]. However, excessive intake produces well-documented harmful effects on health, notably promoting hypertension, and is implicated in the development of kidney, brain, vascular, and immune system diseases [4].

In addition, excess sodium can affect bone tissue independently of vascular effects such as blood pressure increase [5]. Results from animal studies indicate that a high dietary intake of sodium chloride administered for four weeks in growing and adult rats reduces mineral accumulation in bone tissue, an effect primarily attributed to increased urinary calcium excretion and the resulting negative cation balance [6]. Other studies conducted in humans and rats suggest that high-sodium intake is associated with increased urinary excretion of hydroxyproline, a biochemical marker of bone resorption, indicating that sodium intake can impact collagen degradation and bone matrix [7]. Moreover, elevated salt consumption has been associated with a higher risk of fragility fractures in osteoporotic individuals, particularly in the context of low calcium intake and poor adherence to healthy dietary patterns such as the Mediterranean diet, highlighting the critical role of nutrition in bone fragility and skeletal health [8]. Recent evidence has reported that the increased bone resorption associated with high-sodium diets may not only be a consequence of calcium loss in the urine but could also involve direct effects on favoring osteoclast differentiation and increasing their resorptive function, independent of parathyroid hormone (PTH) signaling [9].

Bone homeostasis has traditionally been viewed as a process regulated by endocrine factors such as PTH, growth hormone, and sex steroids. However, over the past two decades, the immune system has emerged as a fundamental regulator of bone remodeling [10]. Clinical observations in inflammatory diseases such as periodontitis and rheumatoid arthritis demonstrate that immune-mediated inflammation accelerates bone loss [11,12]. Mechanistic studies identified key molecules produced by T cells, such as M-CSF and RANK-L, that bridge immune and bone systems [13]. Many immune cell types influence bone cells either directly or via secreted factors like osteoprotegerin, RANK-L, and inflammatory cytokines [14]. Recently, an increased presence of T helper 17 (Th17) cells was observed in the bone marrow of ovariectomized mice, a model of osteoporosis, and these cells were found to enhance the recruitment of inflammatory monocytes as osteoclast precursor cells to the bone marrow [15]. In addition, IL-17, the signature cytokine produced by Th17 cells, promotes bone resorption through several mechanisms. It induces RANK-L secretion by osteoblasts, thereby stimulating osteoclast differentiation, and upregulates RANK expression on osteoclast progenitor cells, enhancing their responsiveness to RANK-L [16].

It has also been demonstrated that excessive salt intake affects the function of regulatory T cells (Tregs), thereby impairing their suppressive capacity [17]. A sustained increase in dietary salt can disrupt the balance between proinflammatory Th17 cells and anti-inflammatory Tregs, favoring Th17 expansion [18]. High-salt conditions promote Th17 differentiation through activation of pathways such as p38/MAPK, NFAT5 and SGK1 [19], and may also influence immune responses through additional molecular and environmental mechanisms, including microbiota modulation and proinflammatory gene programs [20,21], contributing to a proinflammatory lieu. A seminal study by Dar et al. provided key evidence linking high dietary salt intake with bone deterioration through modulation of the Th17–Treg axis. Their findings established that a high-salt diet (HSD) impairs bone microarchitecture, reduces mineral density, and promotes a pro-osteoclastogenic inflammatory environment [22]. However, that study focused exclusively on male mice, employed a single endpoint, and did not integrate complementary analyses of systemic immune responses or bone biochemistry, leaving important aspects of this relationship underexplored.

We hypothesize that chronic high-salt intake induces a dynamic and time-dependent remodeling of immune responses that differentially impacts bone homeostasis over time, contributing to progressive deterioration of bone structure and function. Furthermore, we propose that moderate renal impairment induced by unilateral nephrectomy enhances salt sensitivity, amplifying both immune dysregulation and bone damage.

To address these gaps, we aimed to develop and characterize a murine model to study the effects of a HSD on the chemical and physical properties of long bones, as well as on immune system activation. We implemented unilateral nephrectomy (UNX) to induce moderate renal impairment [23,24], a well-established model of salt sensitivity in rats [25], thereby accelerating susceptibility to salt-induced damage while preserving the possibility of investigating early pathophysiological changes. As discussed for murine models of hypertension [26], mice do not inherently display robust salt-sensitive phenotypes, and reduction in renal reserve provides a controlled strategy to unmask sodium-dependent systemic responses.

This model allows for the longitudinal study of bone and immune alterations under sustained high-salt intake, incorporating both sexes and a comprehensive evaluation of bone physiology (including microarchitecture, composition, and biomechanics) together with immune phenotyping. This integrative approach provides a platform to explore the underlying mechanisms linking sodium overload, immune cells dynamics, and bone integrity.

## 2. Materials and Methods

### 2.1. Mice

Age-matched (7–10 weeks old) mice of both sexes were used. Two mouse strains were employed: C57BL/6J wild-type mice and Foxp3-GFP reporter mice (B6.Cg-Foxp3tm2Tch/J—RRID:IMSR_JAX:006772). Foxp3-GFP reporter mice were purchased from The Jackson Laboratories (USA). All animals were bred in the animal facility of the “Facultad de Ciencias Médicas, Universidad Nacional de Córdoba”, and kept under standard environmental conditions (12 h light/dark cycles, temperature of 20–25 °C, and controlled humidity) with food and water ad libitum. Mice from the same experimental groups were co-housed in cages in groups of 3–6 mice. Foxp3-GFP mice were used to enable accurate identification of regulatory T cells (Tregs) by flow cytometry. In this model, expression of green fluorescent protein (GFP) is driven by the Foxp3 promoter, allowing direct detection of Foxp3^+^ cells without the need for intracellular staining. This approach improves the reliability of Treg quantification and preserves cell integrity for multiparametric analyses. C57BL/6J wild-type mice were included in all experiments except those involving flow cytometry.

### 2.2. Ethics Statement

Mouse handling followed international ethical guidelines. Animal studies were conducted according to the Guide for Care and Use of Laboratory Animals [27]. The protocol (V-25/2022) was approved on 1 September 2022, by CICUAL, the Institutional Committee for Care and Use of Laboratory Animals of the “Facultad de Ciencias Médicas, Universidad Nacional de Córdoba”.

### 2.3. Experimental Design and Animal Treatment

Animals were subjected to unilateral nephrectomy (UNX) [23] under anesthetic induction with a ketamine/xylazine cocktail at doses of 50 mg/kg and 20 mg/kg, respectively, with intraperitoneal injection. After the UNX procedure, all mice were allowed a 30-day recovery period before being randomly assigned to the experimental diets. This stabilization period was strictly implemented to ensure that the acute hemodynamic, endocrine, and inflammatory effects associated with the surgical procedure were resolved, and that animals had achieved a stable physiological state with compensated renal function prior to the introduction of the HSD.

Subsequently, they were divided into two experimental groups: normal sodium diet (NSD) with 0.2% NaCl consisting of standard food pellets (commercial brand GEPSA FEEDS), and high-sodium diet (HSD) with 4% NaCl that was prepared in the following way: NaCl was dissolved in warm water and then atomized onto food pellets, dried at 60 °C, and subsequently used to feed the animals. Thus, experimental diets were administered through food, meaning animals ingested NaCl orally and naturally. The 4% NaCl concentration was selected based on its established efficacy in inducing measurable physiological changes in rodent models with reduced renal reserve [25]. Furthermore, this experimental model has been previously validated by our group to model salt-sensitive metabolic alterations [28], providing a robust experimental tool to investigate mechanisms of sodium-induced damage without compromising animal survival.

The number of animals included in each experimental group was as follows: NSD for 20 days (*n* = 6 females and 6 males), HSD for 20 days (*n* = 6 females and 6 males), NSD for 60 days (*n* = 4 females and 5 males), HSD for 60 days (*n* = 4 females and 6 males), NSD for 150 days (*n* = 4 females and 4 males), and HSD for 150 days (*n* = 5 females and 4 males). All groups completed the expected protocol duration, and no mortality was observed during the dietary intervention. Given the range of experimental procedures, not all parameters were evaluated in every animal, and a subset of animals was randomly selected for specific analyses. The number of samples analyzed for each experiment is specified in the corresponding figure legends.

The animals were placed individually in metabolic cages for 24 h urine collection and water consumption assessment at four time points: first at the beginning of the study (baseline), then at 20, 60, and 150 days post-diet (dpd). Body weight and systolic blood pressure were recorded at baseline, every 15 days, and at the final day of the protocol. Endpoints were set at 20, 60, and 150 dpd. At each time point, animals were anesthetized using 45 µL of isoflurane and blood samples were collected via cardiac puncture. Immediately after, animals were euthanized by cervical dislocation, and tissues were collected for subsequent analyses. Depending on the experimental endpoint, spleen and femur samples were processed for flow cytometry, biomechanical testing, or compositional analyses. Tissues were either placed in PBS containing 2% FBS at 4 °C and processed immediately, or preserved under appropriate conditions (e.g., frozen) until further use (Supplementary Figure S1).

### 2.4. Blood Pressure Measurement

Blood pressure was measured without anesthesia using a noninvasive tail-cuff method (CODA™ Noninvasive Blood Pressure System, Kent Scientific, Torrington, CT, USA), as described in our previous studies [29,30]. In summary, animals were acclimated to the tail-cuff procedure in a quiet room and with the same operator for one week before the start of the protocol. Each mouse was placed in a plastic restrainer maintained at 33–36 °C. An occlusion cuff was inflated around the tail to temporarily restrict blood flow. The cuff was then gradually deflated while a second cuff, utilizing volume pressure recording technology, recorded tail swelling caused by arterial pulsations. Systolic blood pressure (SBP) was automatically determined at the first detection of this swelling. The final SBP value of a recording is expressed as the mean of ten measurements.

### 2.5. Biochemical Parameters Assessment

Serum calcium, phosphorus, and creatinine, as well as urinary calcium and creatinine were measured by spectrophotometry using a Biosystems A25 autoanalyzer (Biosystems S.A., Barcelona, Spain). PTH was measured by ELISA (MicroVue Mouse PTH 1-84 EIA). Urinary sodium, chlorine and potassium were measured by ion-selective electrodes. Additionally, the creatinine clearance was calculated with the following formula: (urinary creatinine (mg/24 h))/(serum creatinine (mg/dL)) × (24 h urine volume (mL))/1440.

### 2.6. Tibial and Femoral Lengths

Prior to any other assessments, the lengths of the long bones were measured using a digital caliper (measuring range: 0–150 mm and resolution: 0.01 mm).

### 2.7. Evaluation of Bone Biomechanical Parameters

Bone biomechanical studies were performed on the right femur, using a 3-point flexion test to evaluate cortical bone [31,32]. Femurs were stored at −20 °C wrapped in saline-soaked gauze until testing and were thawed at 37 °C before analysis. Mechanical testing was conducted using a custom-built device equipped with a 200 N load cell, a resolution of 0.01 N, and a displacement accuracy of 10 μm. The span between the two support bars was set at 8.5 mm, the contact point geometry was slightly rounded, and a constant displacement rate of 0.01 mm/s was used. load–displacement data were recorded using the Biomedical Data Acquisition Suite 1.0 (Argentina, 2011) at a sampling rate of 10 Hz. The ultimate load (N) was defined as the peak load achieved, while the fracture load (N) was recorded just before the initial drop in force. Stiffness (N/mm) was calculated as the slope of the linear portion of the load–displacement curve. Absorbed energy (mJ) was defined as the area under the curve up to the fracture point. Material properties were derived using classical beam theory, converting load and displacement into stress [stress = load × two-bar distance × outer radius/(4 × CSMI) and strain [strain = (12 × outer radius × displacement × 10^6^)/(two-bar distance)^2^]. Ultimate stress (MPa) was defined as the maximum calculated stress, and the elastic modulus or Young’s modulus (GPa) was determined from the linear portion of the stress–strain curve.

### 2.8. Bone Microarchitecture Assessment

Micro-computed tomography (micro-CT) was performed on the left tibia using a SkyScan 1173 device (Bruker, Billerica, MA, USA), with the following settings: voxel size of 6 µm^3^, voltage of 60 kV, current of 133 µA, integration time of 500 ms, and rotation step of 0.2°. After image acquisition, the 1800 projections over 360° were reconstructed using NRecon 1.7.3.2 software (Bruker). Morphometric parameters were measured using Fiji software (version 2.16.0/1.54p) and the BoneJ plugin (version 7.0.20). The volume of interest (VOI) was defined starting 20 slices away from the proximal growth cartilage with a height corresponding to 5% of the total tibia length. Images were filtered with a Gaussian filter (σ = 0.8), segmented with Otsu’s thresholding method, and regions of interest (ROIs) were manually drawn to define the trabecular region. The standard trabecular morphometry parameters analyzed were: trabecular bone volume (BV/TV, %), number of trabeculae (Tb.N, 1/mm), trabecular thickness, (Tb.Th, μm) and trabecular spacing (Tb.Sp, μm) [33,34].

### 2.9. Chemical Composition of Tibia

The right tibias were included in methacrylate and cut in cross sections. The mineral composition and the Ca/P ratio were analyzed on the cortical area by means of scanning electron microscope and energy-dispersive spectroscopy (SEM-EDS) using a Σigma device (Carl Zeiss, Jena, Germany) [35].

### 2.10. Flow Cytometry Analysis

Tissues for flow cytometry were prepared as described elsewhere [36]. Briefly, single-cell suspensions were prepared from spleens by mechanical disaggregation through a tissue strainer. Bone marrow (BM) cells were isolated from femurs after removal of the epiphyses by brief centrifugation at 8000× *g* for 5 s. In both spleen and BM samples, erythrocytes were lysed by incubation with ammonium chloride–potassium phosphate buffer (ACK Lysing Buffer, Gibco, Thermo Fisher, Waltham, MA, USA) for 3 min. Leukocyte counts were subsequently determined using Turk’s solution and a Neubauer chamber.

For surface staining, 2 × 10^6^ cells were incubated in PBS 2% FBS for 20 min at 4 °C with LIVE/DEAD Fixable Aqua Dead Cell Stain Kit, for 405 nm excitation (Invitrogen, Cat# L34966, Thermo Fisher, Waltham, MA, USA) and the following antibodies: PE-Cyanine5.5 anti-CD8 clone 53-6.7 (eBioscience, Cat# 35-0081-82, Thermo Fisher, Waltham, MA, USA), APC-eFluor 780 and Super Bright 645 anti-CD4 (eBioscience, Cat# 47-0041-82 and Cat# 64-0041-82 respectively), Pacific Blue anti-CD4 (Biolegend, Cat# 100428, San Diego, CA, USA), PE-Cyanine7 anti-NK1.1 (eBioscience, Cat# 25-5941-82), PE-Cyanine7 anti-CD25 clone PC61.5 (eBioscience, Cat# 25-0251-82), PE/Dazzle 594 anti-CD39 clone Duha59 (Biolegend, Cat# 143812), PE anti-PD-1, clone RMPI-30 (Biolegend, Cat# 109104), PE-Cyanine5 anti-CD44, clone IM7 (eBioscience, Cat# 15-0441-81), Alexa Fluor 700 anti-CD45 clone 30-F11 (eBioscience, Cat# 56-0451-82), PE anti-IL-17 clone TC11-18H10 (BD Bioscience, Cat# 559502), FITC anti-Foxp3 clone FJK-16s (eBioscience, Cat# 11-5773-82).

To assess intracellular cytokines, cells (2 × 10^6^ per well) were cultured in 200 μL of supplemented RPMI 1640 medium and stimulated for 5 h at 37 °C with PMA (50 ng/mL; Sigma-Aldrich, Cat# P1585, Saint Louis, CA, USA) and ionomycin (1 μg/mL; Sigma-Aldrich, Cat# I0634) in the presence of Brefeldin A (eBioscience, Cat# 00-4506-51) and Monensin (Cat# 00-4505-51). After stimulation, cells were first stained for surface markers, including an anti-Foxp3 antibody, given that GFP fluorescence is lost during permeabilization, then fixed and permeabilized using Foxp3/Transcription Factor Staining Buffers (eBioscience, Cat# 00-5523-00), following the manufacturer’s one-step protocol. Intracellular staining was performed for 30 min at room temperature.

Data acquisition was performed on an LSRFortessa cytometer (BD Biosciences, San Jose, CA, USA), and analyses were carried out using FlowJo software (version X.0.7).

### 2.11. Statistical Analysis

Results are expressed as mean ± SD unless otherwise specified. Data normality was assessed using the Shapiro–Wilk test. Two-way ANOVA was performed to determine the effect of the interaction between sex and diet on main physiological parameters, while three-way ANOVA including sex, diet, and time was used on key bone-related outcomes. Endpoint variables were compared in groups at the same time point using a *t*-test when data followed a normal distribution, or the Mann–Whitney test otherwise. Follow-up variables measured repeatedly in the same animals, such as body weight and systolic blood pressure, were analyzed using repeated measures ANOVA followed by appropriate post hoc tests. Pairwise correlation test was used to evaluate the association between main bone and immune variables. Sample sizes (*n* = 4–6 per group) were determined based on previous studies and validated by post hoc sensitivity power analyses. For a significance level of α = 0.05, a sample size of *n* = 6 provided a statistical power of 0.88 to detect a Cohen’s d effect size of 2.02, while *n* = 4 was sufficient to identify the large biological effect sizes characteristic of this chronic high-salt exposure model.

Depending on the figure, statistical differences are represented either by exact *p* values or by symbols indicating significance thresholds (* *p*  <  0.05, ** *p*  <  0.01, *** *p*  <  0.001), as specified in each case. Differences were considered statistically significant when *p*  <  0.05, and values between 0.05 and 0.1 were interpreted as indicative of a trend. In some cases, as indicated in the figure legends, outliers were identified using the ROUT method. For certain determinations, data were pooled from independent experiments to achieve the required sample size for statistical significance across 1–3 separate assays. The total sample size for each experiment is specified in the figure legends. Statistical analyses and data visualization were carried out using Stata (v17.0) and GraphPad Prism (v9.0).

## 3. Results

### 3.1. HSD Induces a Transient Spike in Systolic Blood Pressure, and an Increase in Water Consumption and Urine Volume

**Figure 1:**
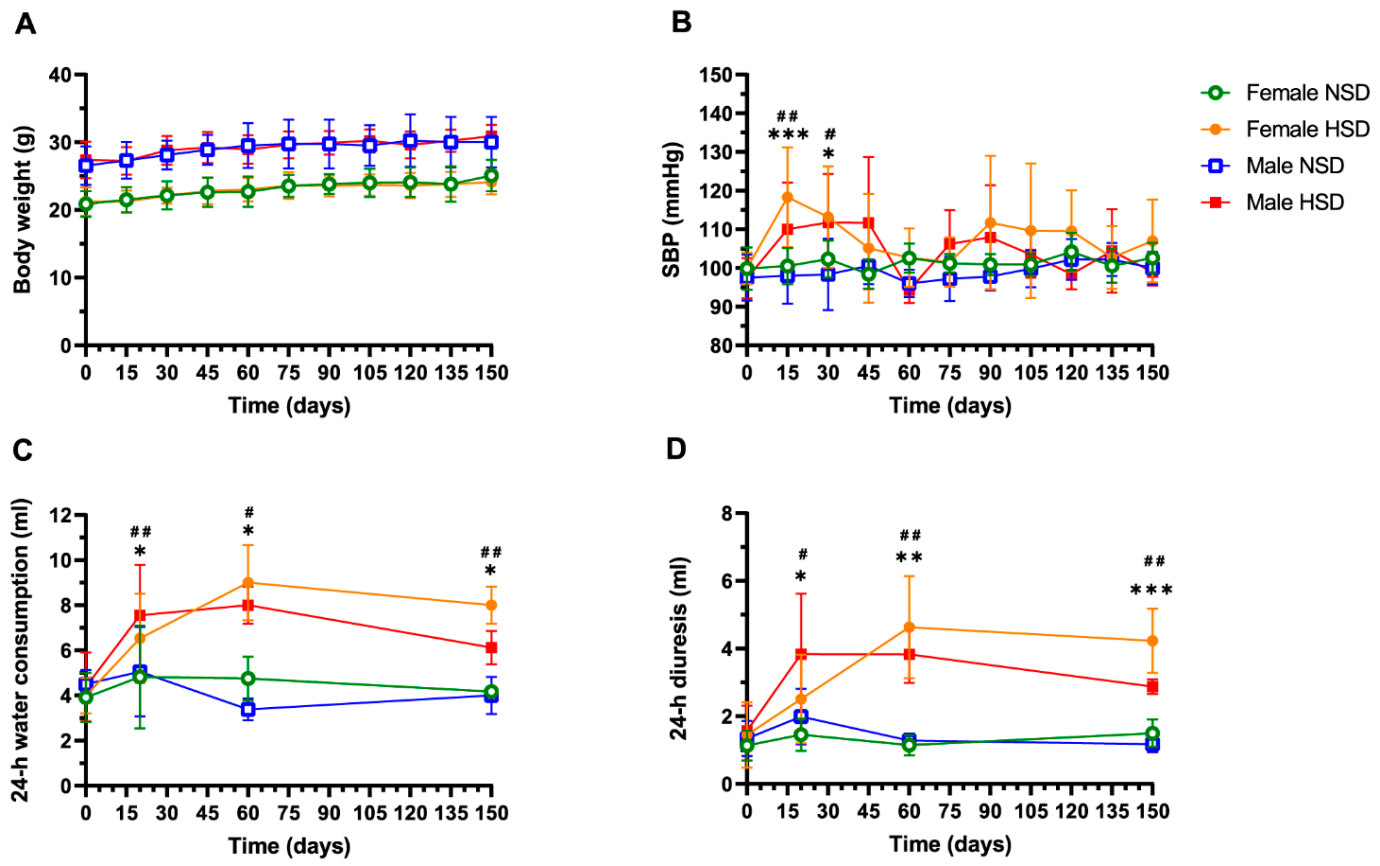
High-salt diet induces a transient elevation in systolic blood pressure and a sustained increase in water consumption and diuresis. Kinetic analysis of physiological parameters in mice fed a normal salt diet (NSD) or a high-salt diet (HSD). (**A**) Body weight, (**B**) systolic blood pressure (SBP), (**C**) 24 h water consumption and (**D**) 24 h urine output. Data are presented as mean ± SD. Data include both C57BL/6 and Foxp3-GFP mice. Female NSD (*n* = 6) vs. HSD (*n* = 6) at 20 days, NSD (*n* = 4) vs. HSD (*n* = 4) at 60 days, NSD (*n* = 4) vs. HSD (*n* = 5) at 150 days: * *p* < 0.05, ** *p* < 0.01, *** *p* < 0.001; male NSD (*n* = 6) vs. HSD (*n* = 6) at 20 days, NSD (*n* = 5) vs. HSD (*n* = 6) at 60 days, NSD (*n* = 4) vs. HSD (*n* = 4) at 150 days: # *p* < 0.05, ## *p* < 0.01; repeated measures ANOVA (**A**,**B**) and *t*-test (**C**,**D**).

To assess the impact of HSD on bone physiology and immune activation, we developed a murine model involving UNX followed by long-term exposure to either HSD or NSD (normal sodium diet), as outlined in Supplementary Figure S1. We first evaluated whether both experimental groups exhibited comparable food intake by monitoring body weight gain over time. As shown in Figure 1A, although females had lower body weight than males, mice in both the HSD and NSD groups displayed similar weight gain, indicating that dietary salt enrichment did not affect weight gain in either sex.

We next evaluated physiological parameters associated with salt overload. As shown in Figure 1B, systolic blood pressure (SBP) increased in the HSD group at 15 and 30 days post-diet (dpd) but returned to baseline by 45 dpd and remained stable thereafter. In line with increased salt intake, HSD-fed animals consumed more water and exhibited higher urine output at all three evaluated time points (20, 60, and 150 dpd), as shown in Figure 1C,D.

Two-way ANOVA analysis revealed significant main effects of diet, as well as sex-dependent differences for some physiological parameters, but no significant sex × diet interaction (Supplementary Table S1), indicating that the effects of dietary salt were comparable between sexes. Accordingly, data are hereafter presented as aggregated results by dietary condition (NSD or HSD), without separation by sex, to highlight the effects of diet and for easier visual representation of the results.

### 3.2. HSD Induces Calciuria and Altered Renal Clearance

**Figure 2:**
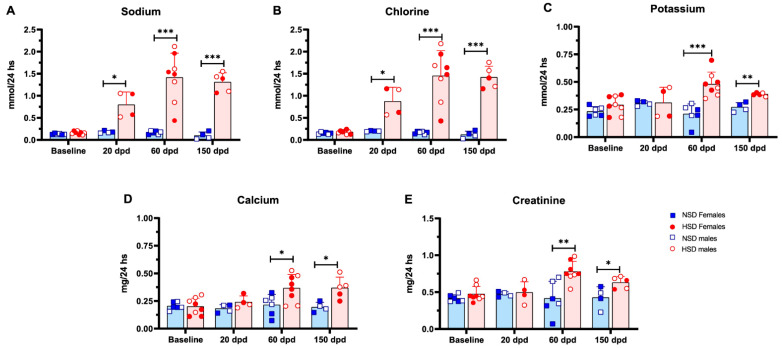
Excess salt in diet rapidly increases sodium and chloride renal excretion, followed by a later increase of potassium, calcium and creatinine. Kinetic analysis of urinary biochemical markers in mice fed a normal salt diet (NSD) or a high-salt diet (HSD). (**A**) Sodium, (**B**) chlorine, (**C**) potassium, (**D**) calcium and (**E**) creatinine at 20, 60, and 150 days post-diet (dpd). Data are presented as mean ± SD, blue bars represent NSD and red bars represent HSD. Data include both sexes and both C57BL/6 and Foxp3-GFP mice. NSD (*n* = 3 female and 3 male) vs. HSD (*n* = 4 female and 4 male) at baseline; NSD (*n* = 2 female and 2 male) vs. HSD (*n* = 2 female and 2 male) at 20 dpd; NSD (*n* = 3 female and 3 male) vs. HSD (*n* = 4 female and 4 male) at 60 dpd; and NSD (*n* = 2 female and 2 male) vs. HSD (*n* = 2 female and 3 male) at 150 dpd. * *p* < 0.05, ** *p* < 0.01, *** *p* < 0.001; *t*-test.

To further characterize the effects of HSD, we analyzed biochemical serum and urine markers related to mineral metabolism and renal function. As expected, 24 h urinary excretion of sodium and chloride was consistently higher in HSD animals at all time points (Figure 2A,B), confirming sustained intake of the NaCl-enriched diet. In addition, HSD mice exhibited increased urinary potassium, calcium and creatinine excretion at 60 and 150 dpd (Figure 2C–E). Despite the rise in calciuria, serum levels of calcium, phosphorus, and PTH remained unchanged throughout the study (Table 1). Serum creatinine was also comparable between groups across all time points. At the start of the dietary intervention, body weight and serum creatinine levels were comparable between groups, confirming a similar baseline physiological status following recovery from surgery. However, creatinine clearance was elevated in the HSD group at 60 and 150 dpd, suggesting glomerular hyperfiltration as a compensatory response to UNX.

### 3.3. HSD Induces Progressive Alterations in Bone Resistance, Mineral Composition, and Microarchitecture

To investigate the cumulative effects of prolonged HSD on bone physiology, we evaluated mechanical performance, mineral composition, and microarchitecture of long bones.

Three-way ANOVA revealed significant main effects of sex, consistent with reported sexual dimorphism on bone parameters, whereas no significant diet × sex interaction was detected (Supplementary Table S2). These results indicate that the impact of HSD on bone was comparable between sexes. Accordingly, data are hereafter presented as aggregated results by dietary condition (NSD or HSD), without separation by sex, to highlight the effects of diet and for easier visual representation of the results.

Mechanical testing by three-point bending revealed that femoral strength was progressively impaired in HSD mice. While no differences were observed at 20 dpd, fracture load and ultimate load were significantly reduced at both 60 and 150 dpd (Table 2). At 150 dpd, additional declines were detected in stiffness, ultimate stress, and Young’s modulus, indicating a loss of structural resistance and material quality. These findings suggest that prolonged HSD compromises the biomechanical performance of the femoral diaphysis.

**Figure 3:**
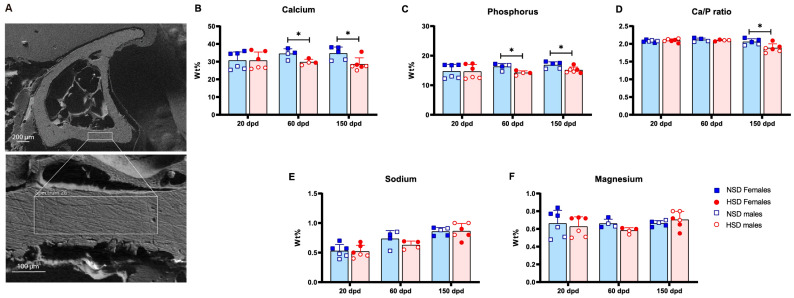
Excess dietary salt impairs bone mineralization. Cortical bone mineral composition in mice fed a normal salt diet (NSD) or a high-salt diet (HSD). (**A**) Representative SEM images of tibial cross-sections and the region selected for elemental analysis. (**B**) Calcium, (**C**) phosphorus, (**D**) Ca/P ratio, (**E**) sodium, and (**F**) magnesium content in the cortical region of the tibia at 20, 60, and 150 dpd, measured by scanning electron microscopy and energy-dispersive spectroscopy (SEM-EDS). Data are presented as mean ± SD, blue bars represent NSD and red bars represent HSD. Data include both sexes and both C57BL/6 and Foxp3-GFP mice. NSD (*n* = 3 female and 3 male) vs. HSD (*n* = 3 female and 3 male) at 20 dpd; NSD (*n* = 2 female and 2 male) vs HSD (*n* = 2 female and 2 male) at 60 dpd; and NSD (*n* = 3 female and 2 male) vs. HSD (*n* = 3 female and 3 male) at 150 dpd. * *p* < 0.05 (*t*-test).

To assess compositional changes, we analyzed the elemental content of cortical bone in the tibia using scanning electron microscopy coupled with energy-dispersive spectroscopy (SEM-EDS). A representative SEM image and the region selected for elemental analysis are shown in Figure 3A. HSD significantly reduced calcium and phosphorus content at 60 and 150 dpd (Figure 3B,C), and decreased the Ca/P ratio at 150 dpd (Figure 3D). Sodium and magnesium content remained unchanged throughout (Figure 3E,F).

**Figure 4:**
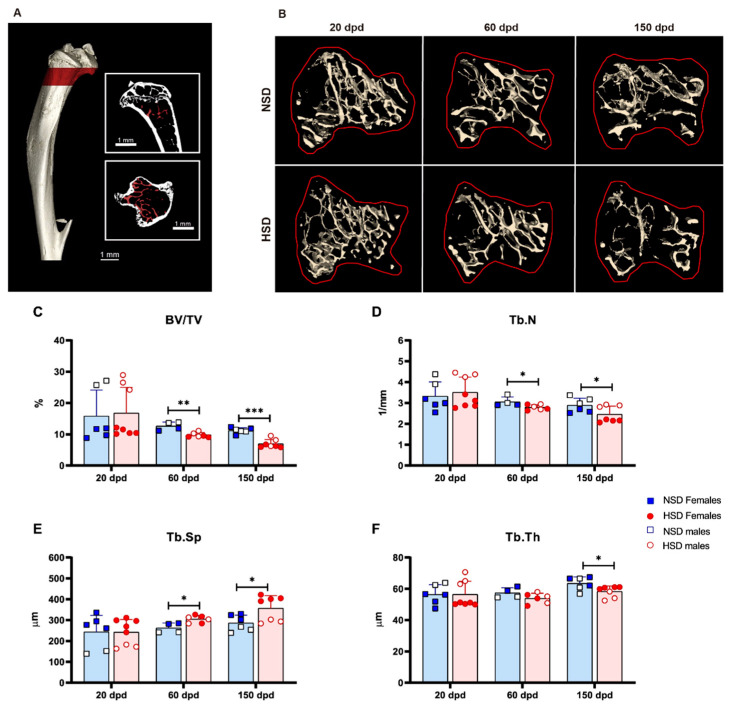
High-salt diet compromises bone microarchitecture. Trabecular bone microarchitecture in mice fed a normal salt diet (NSD) or a high-salt diet (HSD). (**A**) Representative 3D reconstruction of the tibia showing the region of analysis in red, with coronal and transverse views. (**B**) Axial 3D reconstructions of the proximal tibial trabecular region with the analyzed area outlined in red at 20, 60, and 150 dpd of representative NSD and HSD female mice. (**C**) Bone volume fraction (BV/TV), (**D**) trabecular number (Tb.N), (**E**) trabecular separation (Tb.Sp), and (**F**) trabecular thickness (Tb.Th). Data are shown as mean ± SD, blue bars represent NSD and red bars represent HSD. Data include both sexes and both C57BL/6 and Foxp3-GFP mice. NSD (*n* = 4 female and 2 male) vs. HSD (*n* = 5 female and 3 male) at 20 dpd; NSD (*n* = 2 female and 2 male) vs. HSD (*n* = 3 female and 3 male) at 60 dpd; and NSD (*n* = 3 female and 3 male) vs. HSD (*n* = 4 female and 3 male) at 150 dpd. * *p* < 0.05, ** *p* < 0.01, *** *p* < 0.001 (*t*-test).

Micro-CT analysis of the trabecular compartment in the proximal tibia revealed progressive deterioration of bone microarchitecture in HSD mice. The selected VOI is shown in Figure 4A as red areas. As illustrated by the 3D reconstructions (Figure 4B), structural alterations became more evident over time. Bone volume fraction (BV/TV) and trabecular number (Tb.N) were significantly decreased at 60 and 150 dpd (Figure 4C,D), while trabecular separation (Tb.Sp) increased (Figure 4E). Trabecular thickness (Tb.Th) was also reduced at 150 dpd (Figure 4F).

Together, these data indicate that HSD leads to progressive over time multilevel bone deterioration, affecting strength, composition, and trabecular structure.

### 3.4. HSD Induces a Transient Proinflammatory Immune Response Followed by Late Suppression of T Cell Activation

**Figure 5:**
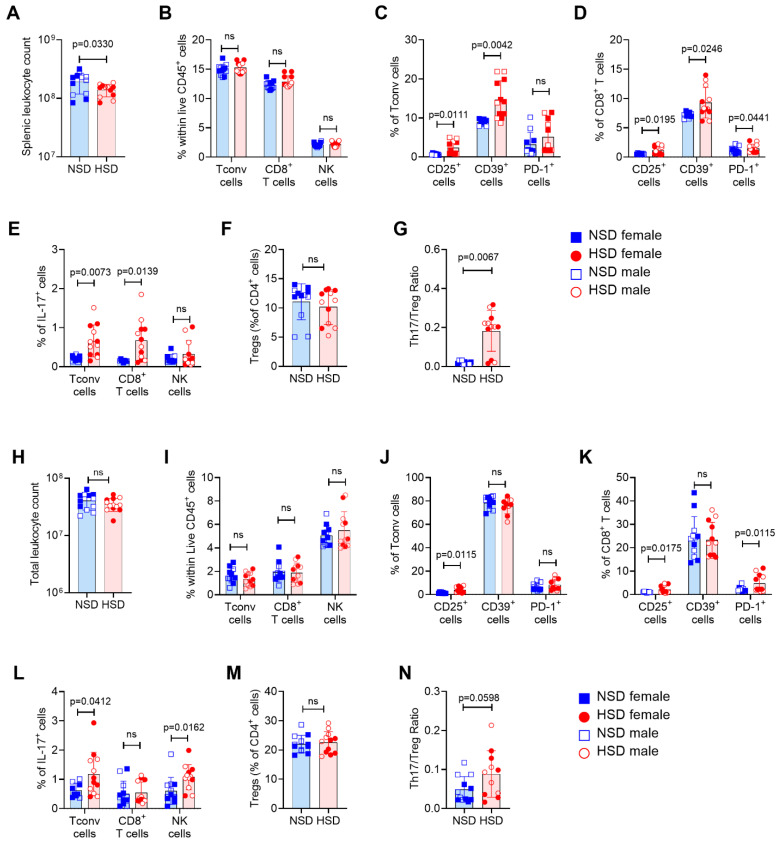
A systemic and local inflammatory response is observed early after high-salt exposure. Immune response evaluation in Foxp3-GFP mice after 20 days of normal salt diet (NSD) or high-salt diet (HSD). Bar graphs showing the distribution of data from spleen (**A**–**G**) and bone marrow (**H**–**N**). (**A**,**H**) Total leukocyte counts. (**B**,**I**) Frequency of conventional T cells (Tconv), CD8^+^ T cells, and natural killer cells (NK) within live CD45^+^ cells. (**C**,**J**) Frequency of CD25^+^, CD39^+^, and PD-1^+^ cells within Tconv. (**D**,**K**) Frequency of CD25^+^, CD39^+^, and PD-1^+^ cells within CD8^+^ T cells. (**E**,**L**) Frequency of IL-17^+^ cells within Tconv, CD8^+^ T cells, and NK. (**F**,**M**) Frequency of regulatory T cells (Treg) within CD4^+^ cells. (**G**,**N**) Th17/Treg cell ratio. Each symbol represents an individual mouse. Females are shown with filled symbols and males with open symbols. NSD is represented by blue squares and HSD by red circles. Data are shown as mean ± SD, blue bars represent NSD and red bars represent HSD. NSD (*n* = 6 female and 6 male) vs. HSD (*n* = 6 females and 6 males) at 20 dpd. Data include both sexes and only Foxp3-GFP mice. Statistical comparisons were performed using unpaired *t* test (**A**–**E**,**G**–**I**,**K**,**M**,**N**) and Mann–Whitney test (**F**,**J**,**L**). Exact *p* values are indicated in the graphs when *p* < 0.05, while *p* > 0.05 is indicated as non-significant (ns).

To evaluate the immunological impact of HSD, we analyzed immune cell populations in the spleen and bone marrow (BM) of Foxp3-GFP reporter mice at different time points. Gating strategy and representative plots are shown in Supplementary Figures S2 and S3. At 20 dpd, splenic leukocyte counts showed a tendency to decrease (Figure 5A), while the frequency of most major immune populations remained unchanged (Figure 5B). However, both Foxp3^−^ CD4^+^ T cells (Tconv) and CD8^+^ T cells exhibited increased activation, as indicated by the upregulation of CD25, CD39, and/or PD-1 (Figure 5C,D). There was also an increase in the frequency of IL-17-producing Tconv (Th17) and CD8^+^ (Tc17) cells but not IL-17-producing NK cells (NK17) (Figure 5E). In addition, there was a trend toward reduced Treg frequency (Figure 5F), resulting in a significantly elevated Th17/Treg ratio (Figure 5G), indicating a shift toward a proinflammatory profile at the expense of regulatory responses.

A similar pattern was observed in BM. Although leukocyte numbers and the frequency of major immune subsets remained unchanged (Figure 5H,I), HSD mice showed higher frequencies of CD4^+^ and CD8^+^ T cells expressing CD25 and/or PD-1 (Figure 5J,K), increased Th17, and NK17 cells (Figure 5L), and conserved Treg frequency (Figure 5M), with a trend toward an elevated Th17/Treg ratio (Figure 5N).

By 60 dpd, the immune alterations observed at 20 dpd were no longer evident. In the spleen, leukocyte counts were significantly reduced (Supplementary Figure S4A), and the frequencies of activated CD4^+^ and CD8^+^ T cells, defined by the expression of CD25, had returned to baseline levels (Supplementary Figure S4B). The frequencies of CD44^+^ effector Tconv and CD8^+^ T cells were also comparable between groups (Supplementary Figure S4C). IL-17-producing Th17 and Tc17 cells, as well as the Th17/Treg ratio, were normalized (Supplementary Figure S4D,E). In the BM, no significant differences were observed in total leukocyte numbers or in the frequencies of activated T cells, CD44^+^ effector cells, or IL-17-producing subsets (Supplementary Figure S4F–J).

**Figure 6:**
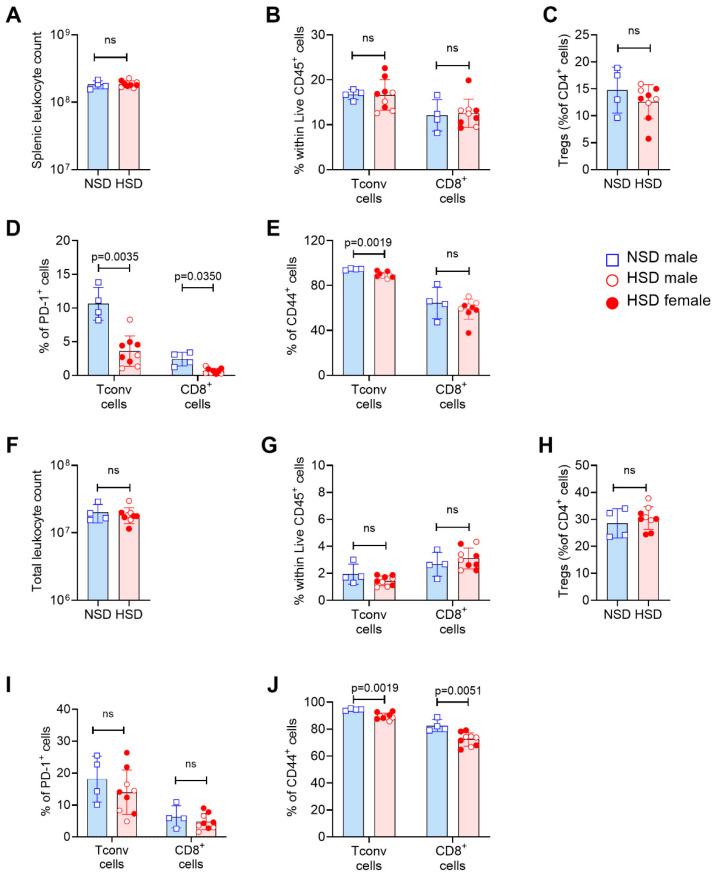
T cell activation and differentiation toward effector phenotypes is diminished after chronic high-salt exposure. Immune response evaluation in Foxp3-GFP mice after 150 days of normal salt diet (NSD) or high-salt diet (HSD). Bar graphs showing the distribution of data from spleen (**A**–**E**) and bone marrow (**F**–**J**). (**A**,**F**) Total leukocyte counts. (**B**,**F**) Frequency of conventional T cells (Tconv) and CD8^+^ T cells within live CD45^+^ cells. (**C**,**H**) Frequency of regulatory T cells (Treg) within CD4^+^ cells. (**D**,**I**) Frequency of PD-1^+^ cells within Tconv and CD8^+^ T cells. (**E**,**J**) Frequency of CD44^+^ cells within Tconv and CD8^+^ T cells. Each symbol represents an individual mouse. Females are shown with filled symbols and males with open symbols. NSD is represented by blue squares and HSD by red circles. Data are shown as mean ± SD, blue bars represent NSD and red bars represent HSD. NSD (*n* = 4 male) vs. HSD (*n* = 5 female and 4 male) at 150 dpd. Data include both sexes and only Foxp3-GFP mice. Statistical comparisons were performed using unpaired *t* test (**A**–**J**). Exact *p* values are indicated in the graphs when *p* < 0.05, while *p* > 0.05 is indicated as non-significant (ns).

At 150 dpd, splenic leukocyte numbers had normalized (Figure 6A), and the frequencies of major immune subsets, including Tconv, Treg, and CD8^+^ T cells, remained similar between groups (Figure 6B,C). Notably, there was a significant reduction in the frequency of PD-1^+^ Tconv and CD8^+^ T cells (Figure 6D), as well as a decrease in CD44^+^ effector Tconv cells (Figure 6E), indicating reduced activation and differentiation of T cells at the systemic level. While the remaining parameters showed no differences between NSD and HSD mice (Figure 6F–J), the frequencies of CD44^+^ effector Tconv and CD8^+^ T cells were also clearly reduced in the BM of HSD mice (Figure 6J). Gating strategy and representative plots are shown in Supplementary Figures S5 and S6.

Of note, correlation analyses revealed that the Th17/Treg ratio in both spleen and bone marrow was inversely associated with trabecular bone volume and microarchitectural indices, including trabecular thickness, and trabecular number, and positively associated with trabecular separation (Table 3). In addition, the Th17/Treg ratio negatively correlated with cortical calcium and phosphorus content.

## 4. Discussion

In recent years, it has become clear that nutrition is one of the most influential modifiable factors affecting bone health. Specific nutrients contribute directly to bone structure or indirectly modulate bone remodeling through effects on calcium absorption and cellular metabolism [37]. In addition, dietary factors can influence immune cell function and inflammation [38], which are increasingly recognized as regulators of bone homeostasis. Excessive dietary sodium, in particular, has been associated with bone demineralization in both human and animal studies [39,40] and immune dysregulation [41]. However, the mechanisms linking high salt intake to bone pathology remain only partially understood, particularly regarding how immune cells influence this process. Some studies have reported impaired bone microarchitecture, altered mineral content, and immune imbalances after short-term high-salt exposure [22]. Our findings are consistent with these observations, including the reported Th17/Treg imbalance, but extend them by providing an integrated and longitudinal perspective on the combined physical, chemical, and immunological effects of chronic dietary sodium overload.

To address these gaps, we established a murine model based on moderate renal mass reduction through UNX followed by sustained high-salt intake, and performed a detailed kinetic analysis of its effects. This strategy allowed us to evaluate the consequences of chronic sodium overload on bone composition, structure, and function across three time points. We also assessed systemic and local immune responses and included both sexes to explore possible hormonal influences.

Importantly, UNX reduces renal mass and impairs sodium excretion [26] while preserving overall function through compensatory mechanisms [42], providing a model of moderate renal impairment that accelerates susceptibility to salt-induced damage [43]. The murine model also offers the advantage of genetic manipulability, enabling mechanistic studies and targeted phenotyping. The use of Foxp3-GFP mice in our experiments allowed detailed flow cytometric characterization of Tregs and other immune populations over time.

In preclinical studies, a diet with 4% NaCl in mice is used as a high-sodium intake model to evaluate the physiological and metabolic effects of excessive salt consumption. As noted by Frieler et al., the salt content used in animal studies far exceeds typical human sodium intake [44]. Nevertheless, this diet reliably induces pathophysiological changes within a short experimental period, allowing us to evaluate the effects of sodium despite its higher concentration compared with the human diet. Our model exhibited only transient elevations in blood pressure, which normalized despite continued dietary intervention. This contrasts with observations in Sprague–Dawley rats fed an 8% NaCl diet, which developed sustained hypertension [45]. In our study, mice exposed to a 4% high-salt diet following UNX showed increased systolic pressure at 15 dpd, but levels remained within the normotensive range throughout the 150-day protocol. This compensatory response aligns with data from other murine models in which C57BL/6 mice exhibit resistance to salt-induced hypertension [46]. Studies in 129S3 mice subjected to subtotal (⅚) nephrectomy demonstrate the importance of strain background in salt sensitivity, further supporting the relevance of our model. In addition to strain selection and nephrectomy, the composition of the salt-loading protocol may influence outcomes. In contrast to studies using 8% NaCl or supplementing drinking water with 1% NaCl [47], we administered a 4% NaCl diet without modifying the drinking water, which may explain the absence of sustained hypertension in our model. In this context, recent work has proposed broadening the definition of salt sensitivity beyond blood pressure responses, incorporating sodium-driven effects on immune cell signaling and cellular metabolism [41]. This expanded view is consistent with our findings, as immune remodeling and bone deterioration occurred in the absence of sustained hypertension.

Body weight gain was comparable between control and HSD groups, consistent with previous reports [42]. Increased urinary calcium excretion was evident in HSD-fed mice, as described in rats by Tiyasatkulkovit et al., and may partially account for the bone damage observed [48]. Sodium and calcium renal reabsorption are partially coupled along the nephron, particularly in the proximal tubule, such that natriuresis is accompanied by increased urinary calcium excretion. This sodium-driven calciuria may contribute to negative calcium balance and promote bone resorption to preserve systemic calcium levels. Interestingly, these alterations occurred in the absence of changes in circulating calcium, phosphorus, or PTH levels, suggesting a direct effect of sodium on bone homeostasis. This hypothesis is supported by in vitro studies showing sodium-induced osteoclastic differentiation and activity independently of PTH [9]. Conversely, increased PTH levels have been reported in ovariectomized rats under salt loading [49] and in young human subjects [50], underscoring the complexity of the underlying regulatory mechanisms. A recent study further demonstrated that sodium can promote PTH secretion in a PiT-1-dependent manner [51], although the in vivo relevance in our model appears limited.

In addition to increased sodium excretion, HSD-fed mice also exhibited elevated urinary potassium and creatinine levels, likely reflecting adaptive renal responses to maintain electrolyte and fluid balance. Increased distal sodium delivery enhances potassium secretion in the collecting duct, a well-established compensatory mechanism in the kidney. High dietary salt intake is also known to modulate the renin–angiotensin–aldosterone system, typically suppressing aldosterone while increasing glucocorticoid activity [52]. Reduced aldosterone levels are consistent with decreased tubular sodium reabsorption and enhanced natriuresis, while increased glucocorticoids may further influence both immune responses and bone remodeling processes. Together, these mechanisms could provide a functional link between renal electrolyte handling, hormonal regulation, and the skeletal alterations observed under chronic high-salt exposure.

Beyond systemic mineral homeostasis, we found that the HSD affected the chemical composition of cortical bone. At 60 and 150 dpd, cortical calcium and phosphorus levels were significantly reduced, and the Ca/P ratio declined at the longest time point. As the mineral component is a key determinant of bone strength and structural integrity [35], these findings are relevant to understanding functional deterioration. Indeed, progressive mineral loss was accompanied by a decline in bone strength as evidenced by biomechanical testing. The correlation between reduced mineral content and diminished mechanical performance has not been previously explored in long bones under salt overload. Our findings contrast with those of Tiyasatkulkovit et al., who reported mechanical impairment only during the first month of salt exposure, which was then resolved [48]. In our model, by contrast, deterioration in bone properties intensified over time, suggesting that chronic exposure to high salt intake can induce cumulative damage to bone tissue. Complementing these observations, micro-CT analysis revealed progressive disruption of trabecular microarchitecture. Bone volume fraction and trabecular number were reduced, while separation increased at both 60 and 150 dpd. A further decrease in trabecular thickness was observed at the later time point, indicating progressive deterioration. These findings are consistent with previous reports [22], but our study provides a more detailed temporal characterization, allowing the observation of progressive structural compromise with prolonged exposure.

Beyond its skeletal effects, HSD also modulated systemic and local immune responses in a time-dependent manner. In line with previous studies reporting Th17/Treg imbalance following salt overload [18], we observed an early proinflammatory profile characterized by increased activation of CD4^+^ and CD8^+^ T cells and expansion of IL-17–producing subsets in both spleen and bone marrow. However, these alterations were transient and followed by a marked reduction in the frequency of PD-1^+^ and CD44^+^ effector T cells at later time points, suggesting a progressive decline in T cell activation and differentiation. This biphasic pattern, marked by early immune activation and subsequent suppression, supports the notion that prolonged HSD may engage compensatory mechanisms aimed at restraining chronic inflammation and highlights a dynamic remodeling of immune cell subsets over time. In line with this temporal profile, Th17-related responses were primarily assessed at early time points, when differences were most evident, whereas later analyses were focused on other aspects of immune remodeling. In this regard, our data indicate that short-term (20 dpd) HSD promotes systemic as well as local (bone) activation of effector T cell subsets, favoring an IL-17–mediated proinflammatory environment, whereas prolonged exposure is associated with a progressive reduction in T cell activation (60 dpd) and a shift toward an overall suppressive immune profile at later stages (150 dpd).

In this context, the association between a proinflammatory immune balance, reflected by an increased Th17/Treg ratio, and impaired bone microarchitecture and mineral content further supports a functional link between immune remodeling and bone deterioration during sustained salt exposure. Although these analyses do not establish causality, they suggest that shifts in the Th17/Treg balance within the bone marrow could alter local cell signaling and immune–stromal interactions, potentially influencing bone cell activity during sustained HSD exposure. Emerging evidence suggests that chronic high-salt intake can modulate the immune system not only through direct effects on T cells but also via indirect mechanisms such as alterations in the gut microbiota, increased glucocorticoid levels, and changes in systemic metabolic mediators, all of which can impact immune function across multiple tissues, including bone [52]. Together, these observations support a model in which immune dysregulation and bone deterioration co-evolve during sustained dietary salt exposure, although the precise mechanisms linking these processes remain to be elucidated. Potential mechanisms may involve SGK1-dependent signaling, which promotes proinflammatory T cell polarization, as well as microbiota–immune interactions that may further shape systemic inflammation and bone remodeling [19,20]

Despite its strengths, this study has limitations that should be acknowledged. To consistently induce salt-sensitive phenotypes, our experimental approach combines chronic high-salt intake with UNX. While this approach effectively reduces renal reserve, it may not fully recapitulate the most common clinical settings of salt sensitivity in humans. Consequently, species-specific variations in renal physiology and sodium handling should be taken into account when translating these findings to human pathophysiology. Nevertheless, this experimental framework enables a controlled environment to investigate sodium-driven systemic, skeletal, and immune alterations within feasible experimental timeframes.

Furthermore, while our analyses reveal robust temporal associations between immune remodeling, particularly changes in the Th17/Treg balance, and bone structural deterioration, they do not establish causal relationships. Further mechanistic studies will be required to determine whether immune dysregulation directly contributes to bone alterations or reflects parallel adaptive responses to sustained salt exposure. In this regard, direct assessment of osteoclastic and osteoblastic activity, together with more detailed characterization of signaling pathways underlying Th17/Treg polarization and the involvement of myeloid cell subsets, may provide further mechanistic insights and help identify potential targets for intervention. Importantly, the model described here provides a platform to explore immune–stromal interactions, bone cell activity, and the signaling pathways that may underlie sodium-associated tissue remodeling under conditions of limited renal reserve.

## 5. Conclusions

Altogether, our results demonstrate that chronic high-salt intake leads to progressive alterations in bone structure, composition, and mechanical properties, accompanied by a dynamic and time-dependent remodeling of immune responses. This process is characterized by an early proinflammatory phase followed by a progressive decline in T cell activation, highlighting a close interplay between immune dysregulation and skeletal alterations under sustained sodium overload. In this context, the murine model described here provides a valuable platform to further investigate the mechanisms linking immune remodeling and bone physiology and to explore potential therapeutic strategies.

## Figures and Tables

**Table 1:** Prolonged high-salt diet alters creatinine clearance. Serum biochemical markers and creatinine clearance in mice fed a normal salt diet (NSD) or a high-salt diet (HSD). Data are presented as mean ± SD. Data include both sexes and both C57BL/6 and Foxp3-GFP mice. NSD (*n* = 2 female and 2 male) vs. HSD (*n* = 2 female and 3 male) at baseline, NSD (*n* = 2 female and 2 male) vs. HSD (*n* = 2 female and 2 male) at 20 dpd, NSD (*n* = 2 female and 2 male) vs. HSD (*n* = 3 female and 2 male) at 60 dpd, NSD (*n* = 2 female and 2 male) vs. HSD (*n* = 2 female and 3 male) at 150 dpd. * *p* < 0.05, ** *p* < 0.01; *t*-test. Creat. Clear: creatinine clearance.

	Baseline		20 dpd		60 dpd		150 dpd	
	NSD	HSD	NSD	HSD	NSD	HSD	NSD	HSD
Ca^+2^ (mg/dL)	10.2 ± 0.3	9.7 ± 0.4	8.4 ± 0.4	8.9 ± 0.3	9.0 ± 0.1	9.5 ± 0.4	9.7 ± 0.5	9.7 ± 0.5
P (mg/dL)	5.9 ± 0.8	5.5 ± 0.8	5.6 ± 0.3	5.9 ± 0.5	5.3 ± 0.1	5.4 ± 0.2	5.4 ± 0.9	5.8 ± 1.4
PTH (pg/mL)	272 ± 189	283 ± 178	210 ± 74	233 ± 105	320 ± 142	316 ± 127	289 ± 123	312 ± 83
Creatinine (mg/dL)	0.41 ± 0.05	0.44 ± 0.08	0.36 ± 0.07	0.33 ± 0.1	0.38 ± 0.05	0.35 ± 0.04	0.38 ± 0.03	0.34 ± 0.04
Creat. Clear. (uL/min)	0.8 ± 0.3	1.4 ± 0.8	1.5 ± 0.3	2.8 ± 1.7	0.9 ± 0.6	8.6 ± 2.3 *	1.0 ± 0.8	4.4 ± 1.5 **

**Table 2:** Sustained high-salt diet weakens biomechanical properties of bones. Femoral mechanical properties in mice fed a normal salt diet (NSD) or a high-salt diet (HSD). Data are presented as mean ± SD. Data include both sexes and both C57BL/6 and Foxp3-GFP mice. NSD (*n* = 4 female and 3 male) vs. HSD (*n* = 5 female and 4 male) at 20 dpd; NSD (*n* = 3 female and 4 male) vs. HSD (*n* = 4 female and 4 male) at 60 dpd, NSD (*n* = 4 female and 4 male) vs. HSD (*n* = 5 female and 4 male) at 150 dpd. * *p* < 0.05, ** *p* < 0.01, *** *p* < 0.001; *t*-test. CSMI: cross-sectional moment of inertia.

	20 dpd		60 dpd		150 dpd	
	NSD	HSD	NSD	HSD	NSD	HSD
External diameter (mm)	1.6 ± 0.17	1.62 ± 0.11	1.71 ± 0.12	1.65 ± 0.14	1.58 ± 0.10	1.66 ± 0.15
Internal diameter (mm)	1.15 ± 0.14	1.15 ± 0.11	1.18 ± 0.11	1.13 ± 0.09	1.05 ± 0.12	1.13 ± 0.12
CSMI (mm^4^)	0.24 ± 0.12	0.25 ± 0.06	0.32 ± 0.08	0.29 ± 0.1	0.26 ± 0.06	0.30 ± 0.11
Fracture load (N)	14.4 ± 2.32	13.2 ± 1.35	12.4 ± 2.98	8.6 ± 1.2 **	16.1 ± 2.7	13.1 ± 1.8 **
Ultimate load (N)	14.5 ± 2.23	13.3 ± 1.35	13.6 ± 1.65	10.1 ± 1.9 **	16.2 ± 2.6	13.3 ± 1.7 **
Stiffness (N/mm)	74.6 ± 32.4	71.5 ± 21.0	71.6 ± 5.64	61.8 ± 12	90.4 ± 16.8	60.8 ± 11 ***
Absorbed energy (mJ)	2.74 ± 0.51	2.21 ± 0.62	1.79 ± 0.53	1.26 ± 0.63	2.41 ± 0.52	2.15 ± 0.74
Ultimate stress (MPa)	106 ± 36.3	93.2 ± 19.1	77.7 ± 15.1	62.3 ± 11 *	108 ± 16.7	79.2 ± 18 ***
Young’s modulus (GPa)	4.2 ± 1.58	3.9 ± 1.27	2.9 ± 0.75	2.8 ± 1.06	5.0 ± 1.27	2.9 ± 1.2 ***

**Table 3:** Pairwise correlation analysis between key bone and immune parameters.

	Spleen Th17/Treg		Bone marrow Th17/Treg	
	r	*p*	r	*p*
BV/TV	−0.9095	0.0905	−0.9921	0.0079
Tb.Th	−0.8865	0.1135	−0.9963	0.0037
Tb.Sp	0.9492	0.0508	0.9715	0.0285
Tb.N	−0.9566	0.0434	−0.9695	0.0305
Cortical Ca	−0.7307	0.0013	−0.7139	0.0019
Cortical P	−0.7286	0.0014	−0.6953	0.0028
Cortical Ca/P ratio	0.0456	0.8668	−0.1045	0.7000
Fracture load	0.3736	0.1396	0.3715	0.1728
Stiffness	−0.1715	0.5105	−0.1536	0.5847
Ultimate stress	0.5211	0.2592	0.5799	0.0235
Young’s modulus	0.2592	0.3324	0.3562	0.2113

## Data Availability

Data are available from the corresponding author upon request.

## Supplementary Materials

The following supporting information can be downloaded at https://www.mdpi.com/article/10.3390/cells15090825/s1, Figure S1: Experimental design; Figure S2: Flow cytometry analysis of splenic immune populations at 20 days of diet; Figure S3: Flow cytometry analysis of bone marrow immune populations at 20 days of diet; Figure S4: No signs of inflammatory immune response are detected after 60 days of high-salt exposure; Figure S5: Flow cytometry analysis of splenic immune populations at 150 days of diet; Figure S6: Flow cytometry analysis of bone marrow immune populations at 150 days of diet; Table S1: Two-way ANOVA analysis of diet and sex across main physiological parameters; Table S2: Three-way ANOVA analysis of time, diet and sex across key bone outcomes.

## Abbreviations

The following abbreviations are used in this manuscript:

BM	Bone marrow
BV/TV	Bone volume fraction
dpd	Days post-diet
HSD	High-Salt Diet
M-CSF	Macrophage colony-stimulating factor
Micro-CT	Micro-Computed Tomography
NSD	Normal Salt Diet
PTH	Parathyroid hormone
RANK-L	Receptor activator of nuclear factor-kB ligand
SBP	Systolic blood pressure
SEM-EDS	Scanning electron microscope and energy-dispersive spectroscopy
Tb.N	Trabecular number
Tb.Sp	Trabecular separation
Tb.Th	Trabecular thickness
Th17	T helper 17 cells
Treg	T regulatory cells
UNX	Unilateral nephrectomy
