# Supplementary material for: A Longitudinal Murine Model Reveals Biphasic T Cell Remodeling and Progressive Skeletal Deterioration Under Chronic High-Salt Exposure
Source: Cells. 2026 May 1;15(9):825. doi: 10.3390/cells15090825 (PMC13162815; doi:10.3390/cells15090825)
Supplement: Supplementary file 1 [file cells-15-00825-s001.zip › cells-4207558-supplementary.pdf]

SUPPLEMENTARY MATERIAL

Supplementary table 1. Two-way ANOVA analysis of diet and sex across main physiological parameters. SBP: Systolic blood pressure

	Diet (p)	Sex (p)	Diet x Sex (p)
Body weight - Baseline	0.233	**<0.001**	0.667
Body weight - 20 dpd	0.667	**<0.001**	0.793
Body weight - 60 dpd	0.862	**<0.001**	0.548
Body weight - 150 dpd	0.968	**<0.001**	0.193
SBP - Baseline	0.971	**0.024**	0.926
SBP - 20 dpd	**<0.001**	**0.015**	0.19
SBP - 60 dpd	0.678	**<0.001**	0.622
SBP - 150 dpd	0.187	**0.005**	0.078
24-h diuresis - Baseline	0.181	0.415	0.842
24-h diuresis - 20 dpd	**<0.001**	**0.022**	0.307
24-h diuresis - 60 dpd	**<0.001**	0.487	0.346
24-h diuresis - 150 dpd	**<0.001**	**0.006**	0.067
24-h water consumption - Baseline	0.964	0.082	0.735
24-h water consumption - 20 dpd	**0.001**	0.315	0.529
24-h water consumption - 60 dpd	**<0.001**	0.054	0.744
24-h water consumption - 150 dpd	**<0.001**	**0.009**	**0.026**

Supplementary table 2- Three-way ANOVA analysis of time, diet and sex across key bone outcomes

	Time (p)	Diet (p)	Sex (p)	Diet x time (p)	Diet x Sex (p)
BV/TV	**<0.001**	**<0.001**	**<0.001**	**<0.001**	0.475
Tb.Th	0.193	0.070	**0.003**	0.065	0.340
Tb.Sp	**<0.001**	**<0.001**	**<0.001**	**0.010**	0.745
Tb.N	**<0.001**	**0.002**	**<0.001**	**0.018**	0.963
Cortical Ca	**<0.001**	**<0.001**	**<0.001**	**0.002**	0.566
Cortical P	**<0.001**	**<0.001**	**<0.001**	**0.002**	0.8992
Cortical Ca/P	**<0.001**	**0.031**	**<0.001**	**0.038**	0.199
Fracture load	**<0.001**	**<0.001**	0.304	0.266	0.358
Stiffness	0.289	0.107	0.379	0.477	0.384
Ultimate stress	**0.026**	**0.023**	0.571	0.961	0.979
Young's modulus	0.192	0.139	0.605	0.278	0.294

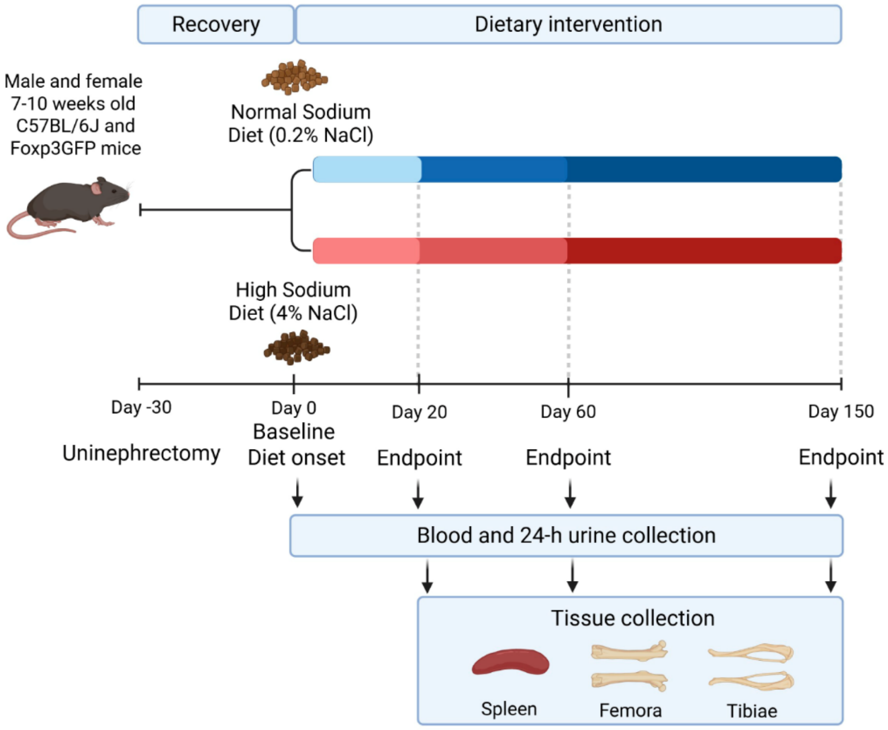

Supplementary Fig. 1 Experimental design. Created with BioRender.com

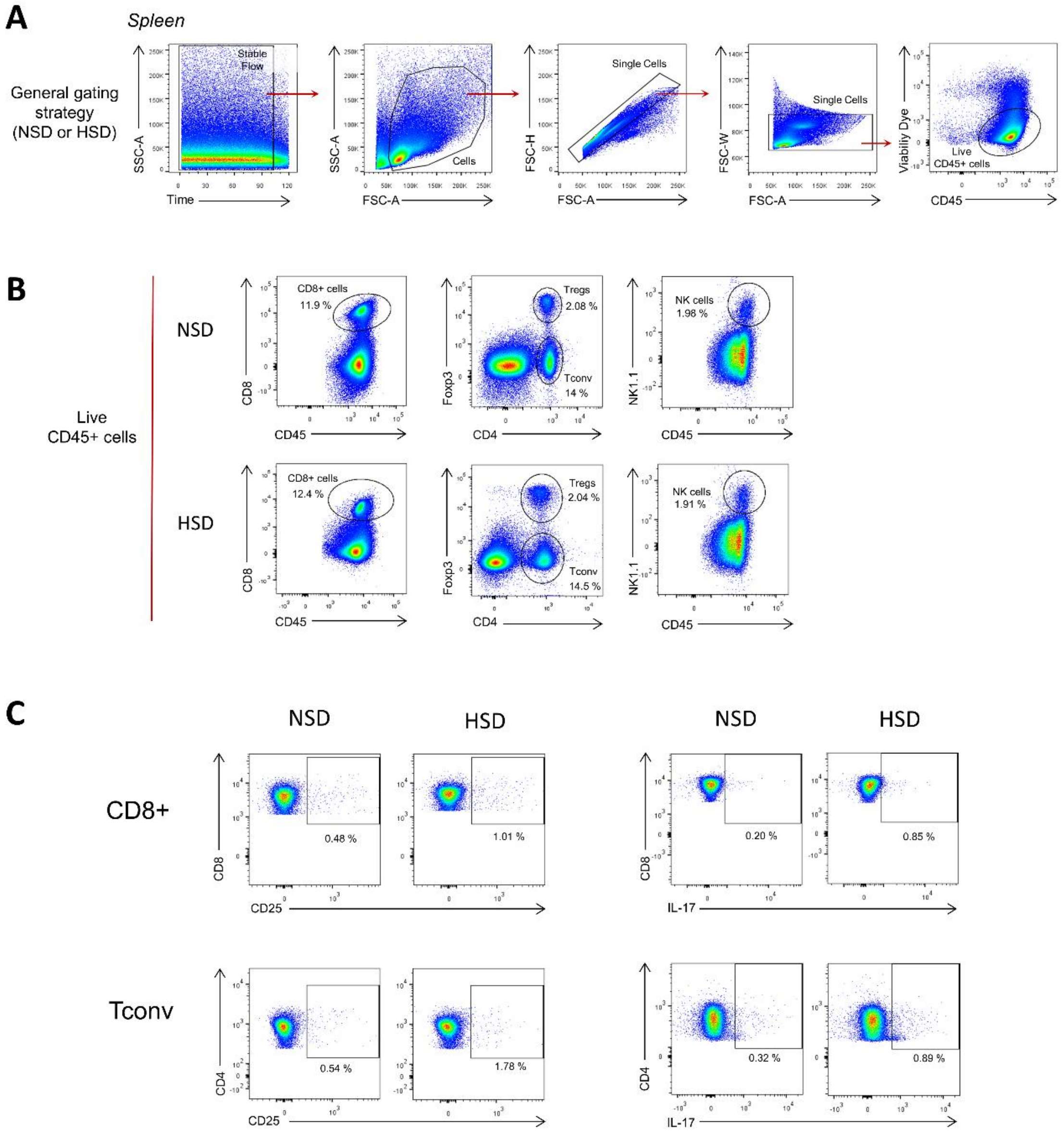

Supplementary Fig. 2. Flow cytometry analysis of splenic immune populations at 20 days of diet. (A) Gating strategy. Events were selected from stable flow regions and based on size and granularity, followed by doublet exclusion and selection of live CD45$^+$ cells. **(B)** Identification of immune cell subsets within live CD45$^+$ cells, including CD8$^+$ T cells, conventional CD4$^+$ T cells (Tconv), regulatory T cells (Treg), and NK cells. **(C)** Representative plots showing CD25 and IL-17 expression within CD8$^+$ and Tconv populations. Dot plots are representative of Foxp3-GFP mice under normal salt diet (NSD) or high salt diet (HSD) at 20 days post diet (dpd).

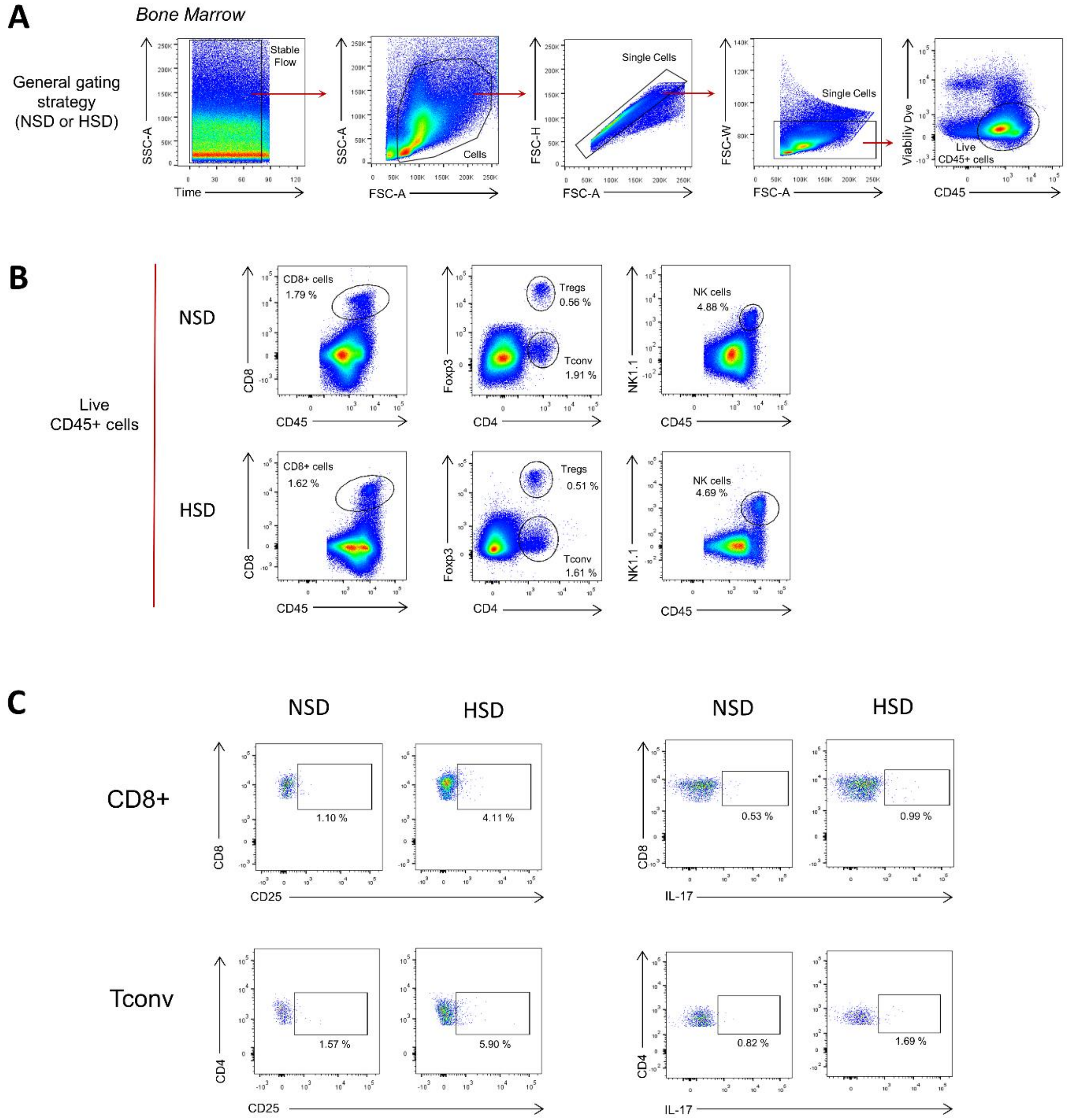

Supplementary Fig. 3. Flow cytometry analysis of bone marrow immune populations at 20 days of diet. (A) Gating strategy. Events were selected from stable flow regions and based on size and granularity (FSC-A vs SSC-A), followed by doublet exclusion and identification of single cells. Live $CD45^+$ cells were then gated for further analysis. **(B)** Identification of immune cell subsets within live $CD45^+$ cells, including $CD8^+$ T cells, Tconv, Treg ($Foxp3^+$), and NK cells. **(C)** Representative plots showing CD25 and IL-17 expression within $CD8^+$ and Tconv populations. Dot plots are representative of Foxp3-GFP mice under normal salt diet (NSD) or high salt diet (HSD) at 20 dpd.

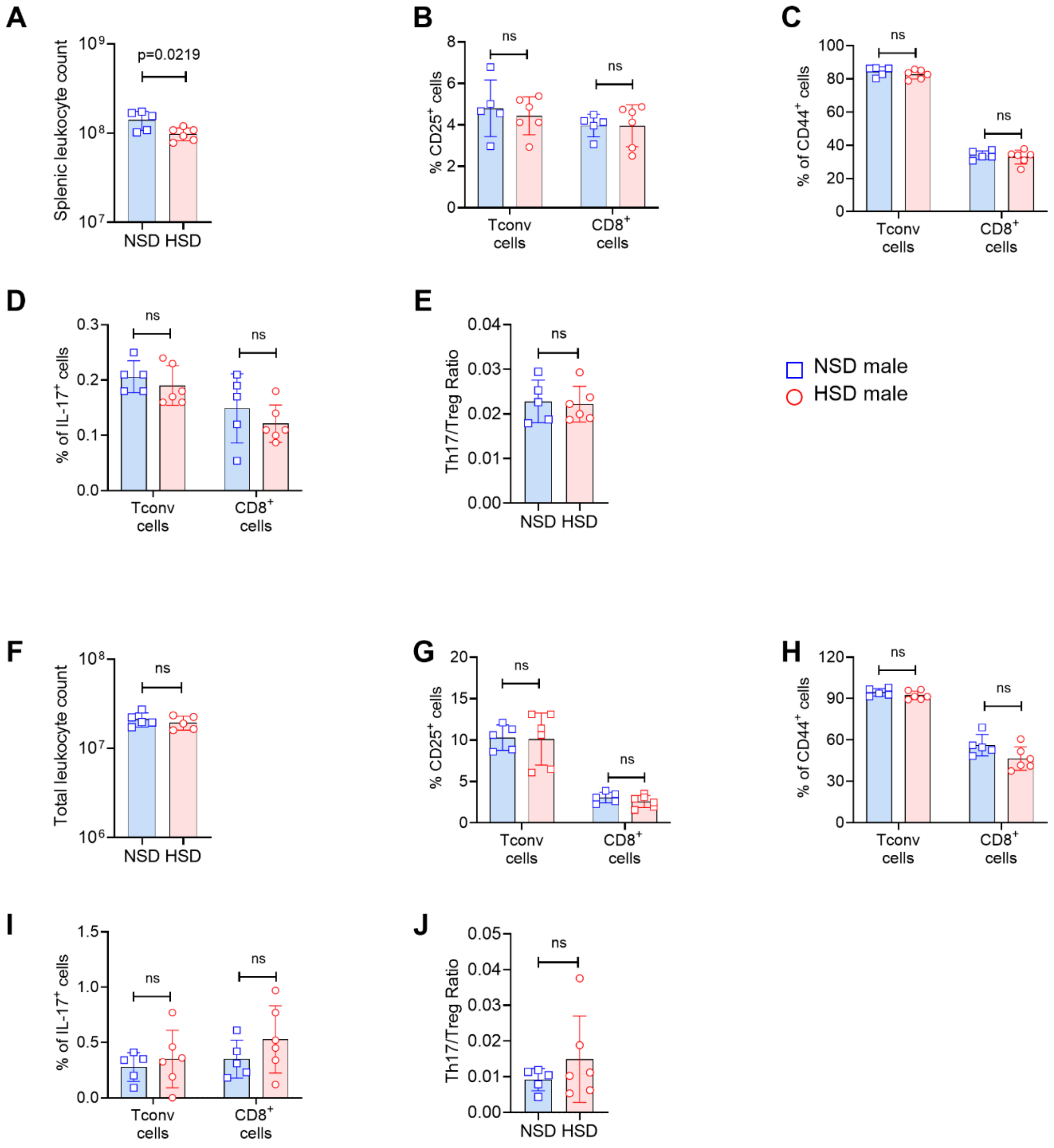

Supplementary Fig. 4. No signs of inflammatory immune response are detected after 60 days of high-salt exposure. Immune response evaluation in male Foxp3-GFP mice after 60 days of normal salt diet (NSD) or high salt diet (HSD). Bar graphs showing the distribution of data from spleen (A–E) and bone marrow (F–J). (A, F) Total leukocyte counts. (B, G) Frequency of $CD25^{+}$ cells within conventional T cells (Tconv) and $CD8^{+}$ T cells. (C, H) Frequency of $CD44^{+}$ cells within Tconv and $CD8^{+}$ T cells. (D, I) Frequency of IL-17^{+} cells within Tconv and $CD8^{+}$ T cells. (E, J) Th17/Treg cell ratio. Each symbol represents an individual mouse. NSD is represented by blue squares and HSD by red circles. Data are shown as mean ± SD, blue bars represent NSD and red bars represent HSD. NSD (n = 5 male) vs. HSD (n = 6 males) at 60 dpd. Data include only Foxp3-GFP mice. Statistical comparisons were performed using unpaired t test (A–J). Exact p values are indicated in the graphs when p<0.05, while p>0.05 is indicated as non-significant (ns).

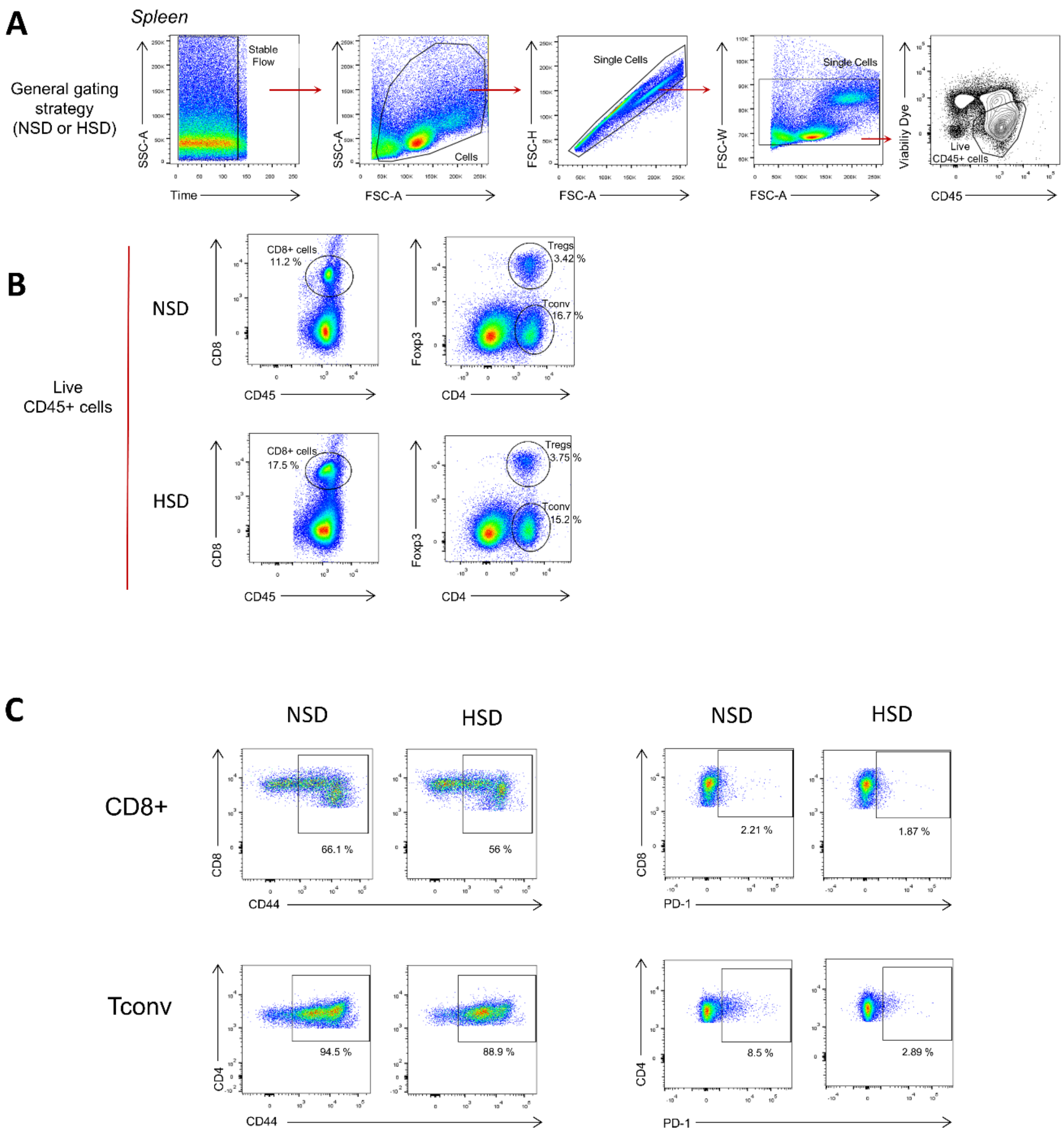

Supplementary Fig. 5. Flow cytometry analysis of splenic immune populations at 150 days of diet. (A) Gating strategy. Events were selected from stable flow regions and based on size and granularity, followed by doublet exclusion and selection of live CD45⁺ cells. **(B)** Identification of immune cell subsets within live CD45⁺ cells, including CD8⁺ T cells, conventional CD4⁺ T cells (Tconv), regulatory T cells (Treg), and NK cells. **(C)** Representative plots showing CD44 and PD-1 expression within CD8⁺ and Tconv populations. Dot plots are representative of Foxp3-GFP mice under normal salt diet (NSD) or high salt diet (HSD) at 150 days post diet (dpd).

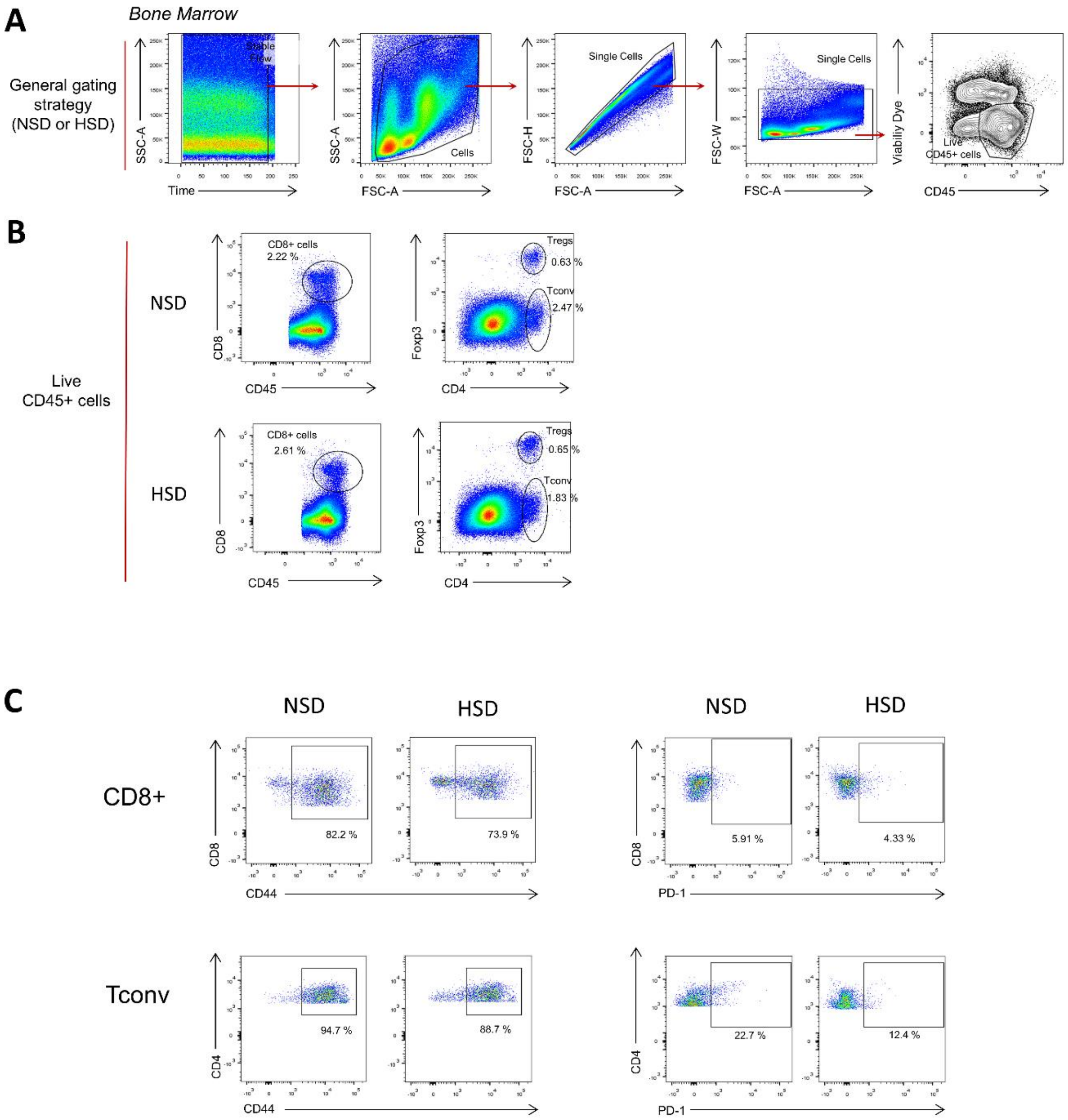

Supplementary Fig. 6. Flow cytometry analysis of bone marrow immune populations at 150 days of diet. (A) Gating strategy. Events were selected from stable flow regions and based on size and granularity (FSC-A vs SSC-A), followed by doublet exclusion and identification of single cells. Live CD45⁺ cells were then gated for further analysis. **(B)** Identification of immune cell subsets within live CD45⁺ cells, including CD8⁺ T cells, Tconv, Treg (Foxp3⁺), and NK cells. **(C)** Representative plots showing CD44 and PD-1 expression within CD8⁺ and Tconv populations. Dot plots are representative of Foxp3-GFP mice under normal salt diet (NSD) or high salt diet (HSD) at 150 days post diet (dpd).
